# Onco-Hypertension: A Continuously Developing Field between Cancer and Hypertension

**DOI:** 10.3390/ijms25063442

**Published:** 2024-03-19

**Authors:** Stefan Totolici, Ana-Maria Vrabie, Elisabeta Badila, Emma Weiss

**Affiliations:** 1 Cardio-Thoracic Pathology Department, “Carol Davila” University of Medicine and Pharmacy, 050474 Bucharest, Romania; stefan.totolici@drd.umfcd.ro (S.T.); elisabeta.badila@umfcd.ro (E.B.); emma.weiss@umfcd.ro (E.W.); 2 Cardiology Department, Colentina Clinical Hospital, 020125 Bucharest, Romania

**Keywords:** secondary hypertension, cardio-oncology, onco-hypertension, antineoplastic therapy, chemotherapy, antihypertensive medication

## Abstract

The prognosis of cancer patients has greatly improved in the last years, owing to the development of novel chemotherapeutic agents. However, this progress comes with an increasing occurrence of cardiovascular adverse reactions. A serious side effect is arterial hypertension (HT), which is the most frequent comorbidity encountered in cancer patients, influencing the outcomes in cancer survivors. Even though secondary HT related to specific chemotherapeutic agents, such as vascular endothelial growth factor inhibitors, is usually mild and reversible, in rare instances it can be severe, leading to discontinuation of chemotherapy. In addition, HT per se has been studied as a potential risk factor for cancer development. The relationship is even more complex than previously thought, as concerning evidence recently highlighted the potential oncogenic effects of antihypertensive drugs, particularly thiazide diuretics, which may increase the risk of skin cancer. As a result, in light of the similar risk factors and overlapping pathophysiological mechanisms between HT and cancer, a promising concept of onco-hypertension has emerged, aiming to improve the understanding of the complicated interplay between these two pathologies and maintain a balance between the efficacy and risks of both antihypertensive drugs and chemotherapy agents.

## 1. Introduction: Cancer and Hypertension

**Figure 1:**
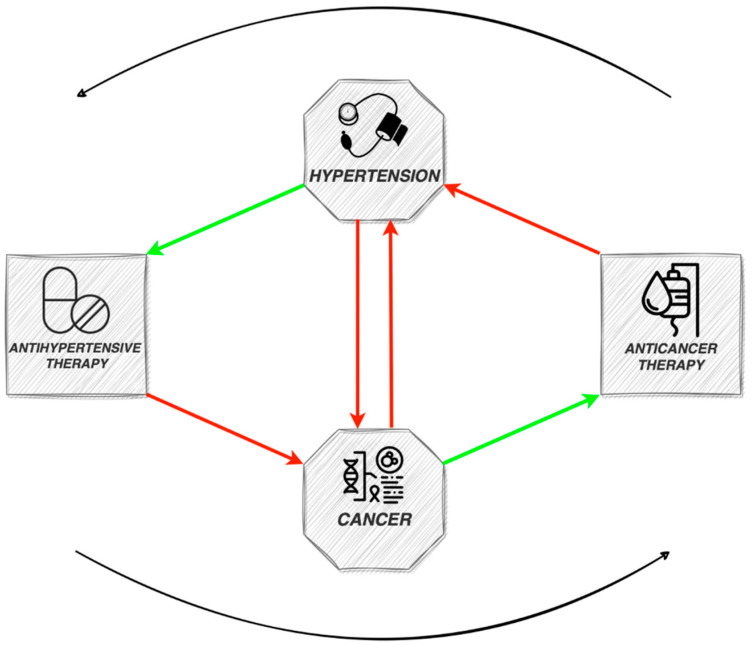
Schematic diagram of the interplay between cancer, anticancer therapy, hypertension and antihypertensive therapy.

In the developed world, cardiovascular diseases (CVD) and cancer stand out as the primary contributors to morbidity and mortality. The relationship between them is complex, as they exhibit a multitude of shared, potentially amendable risk factors, encompassing elevated body mass index, diabetes and tobacco consumption. Hypertension (HT) is a frequent comorbidity in cancer patients and can influence the outcomes in cancer survivors. A noteworthy observation is that the majority of these risk factors are also linked with the onset of HT [1]. The consistent co-occurrence of cancer and HT, alongside shared risk factors, indicates common pathophysiological mechanisms, such as inflammation and increased oxidative stress [1]. However, HT itself has been studied as a potential risk factor for cancer development. As new antineoplastic therapies are constantly emerging, improving the survival rate of cancer patients, the cardiovascular (CV) side effects become even more significant [2]. Although usually secondary HT related to specific chemotherapeutic agents is mild and reversible, in rare instances it can be severe, leading to discontinuation of cancer treatment and affecting prognosis. In addition, serious concerns have lately emerged regarding the potential carcinogenic effects of certain antihypertensive medications. The emerging concept of onco-hypertension has gained more interest, aiming to study the complex interplay between these two entities and ultimately improve the management and outcomes for patients (Figure 1).

## 2. Hypertension Burden in Cancer Patients

CVD represent a leading cause of morbi-mortality in cancer survivors. Much of the increased risk of developing CVD may be attributed to HT as an adverse modifying risk factor. The prevalence of HT in childhood cancer survivors is estimated around 70% by the age of 50 years, significantly greater when compared with the general population [3]. The development of HT in this category contributes to a higher overall CV risk and increased CV-related mortality as compared to cancer patients who remain normotensive. HT in cancer patients may be related to either the type of cancer or, more often, to chemotherapy agents. A strong association between HT and several cancer subtypes, such as renal cell carcinoma, prostate and colorectal cancer has been suggested [4,5].

In adult cancer survivors, HT along other CV risk factors, such as diabetes mellitus, dyslipidemia and obesity, significantly increase the risk of developing coronary artery disease, heart failure, valvular disease and arrhythmia [6]. This risk is subsequently increased after administration of antineoplastic treatment with known cardiotoxic effects, but also after radiotherapy in a dose-dependent manner. Unsurprisingly, the risk of developing CVD is increased when multiple CV risk factors are associated [6]. As a result, early diagnosis of HT and initiation of antihypertensive treatment are crucial in this category of patients in order to improve their survival.

## 3. Anticancer Therapy and Hypertension

In recent years, the progress made in the oncology field has massively improved the survival rate for patients with cancer. However, these new antineoplastic therapies are associated with a series of short- and long-term adverse CV toxicities [1]. A frequent side effect of these drugs is HT, which can arise in patients undergoing diverse chemotherapy types due to direct effects or indirectly through kidney-related mechanisms [7,8]. The risk of cancer therapy-related CV toxicity (CTR-CVT) is subject to variation depending on factors such as the type and stage of cancer, the specific anticancer drugs employed, administered doses, and the presence of underlying comorbidities [9]. HT is widely acknowledged as the primary CV factor that contributes to individuals’ susceptibility to CTR-CVT [10]. Additional therapeutic measures, such as the use of corticosteroids, calcineurin inhibitors, and nonsteroidal anti-inflammatories, along with anti-androgen hormone therapy, may further elevate blood pressure (BP) in individuals undergoing such treatments. Cancer therapy-induced HT is frequently dose limiting and typically exhibits reversibility upon the interruption or cessation of the respective treatment [7].

Recognition of HT induced by antineoplastic agents primarily emerged subsequent to the advent of vascular endothelial growth factor inhibitors (VEGFI), a class of drugs significantly linked to HT in a substantial number of recipients. The documented incidence of VEGFI-related HT spans from 20% to 90%, contingent upon the potency and dosage of the VEGFI administered [7]. Within a meta-analysis encompassing 29,000 cancer patients, the relative risk for HT was notably elevated, presenting a 3.8-fold increase in those subjected to treatment with a vascular endothelial growth factor (VEGF) tyrosine kinase inhibitor (TKI) when compared to their control counterparts [11]. VEGFI finds application as an anticancer treatment across a broad spectrum of malignancies, with a particular emphasis on its use in the metastatic context [1]. The anticancer effects of VEGF signaling pathway inhibitors (VSPIs) manifest through the inhibition of VEGF-mediated tumor angiogenesis. This process results in the deprivation of tumor cells from oxygen and nutrient supply [7]. There are four distinct major classes of agents designed to inhibit VEGF signaling: monoclonal antibodies targeting circulating VEGF; soluble decoy receptors (VEGF-traps) that scavenge freely available VEGF; monoclonal antibodies directed against the vascular endothelial growth factor receptor (VEGFR); and tyrosine kinase inhibitors (TKIs) possessing anti-VEGFR activity, which operate on the intracellular tyrosine kinase domains of VEGFR to impede their activation [1]. While the precise mechanism behind VEGFI-induced HT remains not fully elucidated, several potential mechanisms have been postulated. These include oxidative stress and endothelial dysfunction, along with an altered equilibrium between vasodilators (specifically, a reduction in nitric oxide and prostacyclin I) and vasoconstrictors (manifesting as an increase in endothelin). Other proposed mechanisms encompass vascular remodeling, capillary rarefaction and a reduction in renal sodium excretion. Furthermore, VEGFI have the potential to induce autonomic system toxicity and sympathetic dysregulation leading to the development of HT [12]. These agents induce a rapid and sustained elevation in BP in the majority of patients throughout the course of treatment. The HT associated with VSPIs is characterized by its reversibility, resolving upon discontinuation of the agent. This suggests an on-target effect, signifying that the increase in BP values is attributable to the same mechanisms through which these inhibitors exert their anticancer effects [7,10]. According to the European Society of Cardiology (ESC) guideline on cardio-oncology, it is recommended to conduct daily home BP monitoring during the initial cycle, subsequent to each escalation in anticancer therapy dosage, and at intervals of 2–3 weeks thereafter. Anticipating a decrease in BP upon cessation of VEGFI treatment, adjustments in BP-lowering therapy should be made, including reduction and/or interruption as deemed necessary [9].

Platinum-based compounds are commonly utilized in the treatment of various cancers such as testicular, ovarian, colorectal, bladder, lung cancers, and mesothelioma [1]. The cytotoxic impact demonstrated by these agents arises from the integration of platinum into DNA, culminating in the induction of apoptotic cell death [7]. The HT linked to platinum therapy diverges from that associated with VEGFI, potentially emerging several years following the completion of treatment [1]. Even after a span of 13 years following drug exposure, detectable levels of cisplatin remain in circulation [7]. This holds particular relevance in the context of testicular cancer, which exhibits a high survival rate and stands as the most prevalent cancer among young men. The results of a study involving 1289 survivors of testicular cancer indicated that 53% of individuals who received a cumulative cisplatin dose surpassing 850 mg developed HT over a median follow-up period of 11 years. The occurrence of platinum-associated HT is believed to be significantly influenced by both endothelial cell activation and damage [13].

Proteasome inhibitors, employed in the management of multiple myeloma and mantle cell lymphoma, induce toxicity in malignant cells by binding to the 20S proteolytic core of the proteasome, inhibiting its catalytic activity, and resulting in the intracellular accumulation of aggregated proteins. This also leads to detrimental CV effects, encompassing endothelial dysfunction and diminished bioavailability of nitric oxide [7]. Proteasome inhibitors have been noted for their prohypertensive effects, as evidenced by a trial where HT occurred in 32% of patients treated with carfilzomib compared to 10% in those receiving bortezomib [14]. The vascular effects are contingent on the dosage and duration of treatment [1]. As per the European Society of Cardiology guideline on cardio-oncology, it is recommended to measure BP during every clinical visit for patients undergoing treatment with proteasome inhibitors. Additionally, home monitoring of BP on a weekly basis during the initial 3 months and subsequently on a monthly basis should be considered [9].

PARP (Poly ADP Ribose Polymerase) inhibitors have received approval for utilization in breast and ovarian malignancies. At sites of DNA damage, PARP inhibitors trap PARP1 and PARP2, hindering the recruitment of additional DNA repair proteins. This impediment to DNA repair during tumor cell replication results in apoptosis and subsequent cell death. Among drugs in this class, prohypertensive effects have been observed exclusively with niraparib [1]. A randomized controlled trial reported HT development in 19% of patients treated with niraparib, in comparison to 5% in patients receiving a placebo [15]. The prohypertensive effects attributed to niraparib may signify an off-target effect, particularly the inhibition of the kinase DYRK1A. Such inhibition could potentially elevate levels of neurotransmitters within the dopaminergic system [16].

Bruton Tyrosine Kinase (BTK) inhibitors find application in the therapeutic management of B-cell disorders, such as chronic lymphocytic leukemia, mantle cell lymphoma, Waldenström macroglobulinemia, and marginal zone lymphoma. BTK, an essential and proximal element in B-cell receptor (BCR) signaling pathways, holds a crucial role in the processes of B-cell differentiation, proliferation, and survival. Patients treated with both first- and second-generation BTK inhibitors exhibit an augmented risk for HT development. This risk persists throughout the course of therapy in a cumulative manner [17,18]. Acalabrutinib (second-generation) was compared with ibrutinib (first-generation) in an open label, randomized, noninferiority phase 3 study involving 533 patients with relapsed chronic lymphocytic leukemia. The incidence of any-grade HT was found to be 9.4% in patients treated with acalabrutinib, in contrast to 23.2% in those receiving ibrutinib [19]. Notably, a similar crude incidence of HT was observed in two randomized phase 3 trials comparing zanubrutinib (second-generation) and ibrutinib [20,21]. The comparable incidence of HT across both first- and second-generation BTK inhibitors suggests the presence of a class effect [18]. The mechanisms underlying HT associated with Bruton Tyrosine Kinase inhibitors (BTKIs) are not fully understood, yet a potential significance is attributed to a decrease in heat shock protein 70 signaling and the inhibition of phosphatidylinositol 3-kinase-dependent nitric oxide production [7]. In adherence to the guideline provided by the European Society of Cardiology in the field of cardio-oncology, it is recommended to conduct BP measurements during every clinical visit for patients receiving Bruton Tyrosine Kinase (BTK) inhibitors. Furthermore, weekly home monitoring of BP for the initial 3 months and subsequently on a monthly basis should be considered [9].

Concomitant administration of adjunctive therapies, such as corticosteroids, erythropoietin (EPO), nonsteroidal anti-inflammatory drugs, calcineurin inhibitors or radiotherapy, with antineoplastic agents is a frequent practice. They have the potential to induce HT or aggravate pre-existing controlled HT. The enhanced effectiveness of certain antineoplastic agents is facilitated by corticosteroids, which concurrently alleviate treatment-associated side effects. However, the administration of corticosteroids is associated with notable side effects, including a substantial rise in BP resulting from mineralocorticoid receptor stimulation and subsequent water and sodium reabsorption. Used routinely to manage anemia resulting from the underlying malignancy or anticancer therapy, erythropoietin (EPO) manifests prohypertensive effects. The mechanisms contributing to EPO-induced HT encompass an elevation in blood viscosity and a potential imbalance between vasoconstrictor and vasodilator factors. The well-documented prohypertensive effects of analgesic nonsteroidal anti-inflammatory drugs are modest. The underlying mechanisms for these effects are thought to involve water and salt retention, along with a reduction in the production of vasodilatory prostaglandins [1]. Following hematopoietic stem cell transplantation, calcineurin inhibitors are administered to prevent or address graft versus host disease. These inhibitors have been observed to activate both the renin–angiotensin–aldosterone system and the sympathetic nervous system, resulting in heightened levels of endothelin-1 and reactive oxygen species, coupled with a reduction in nitric oxide. Collectively, these mechanisms contribute to an increased susceptibility to HT [7]. Finally, the occurrence of HT has been correlated with radiation therapy, and the mechanisms contributing to its development may exhibit site-specific characteristics. Specifically, abdominal radiation is seldom associated with renal artery stenosis, while head and neck radiation can induce labile HT by disrupting the baroreflex [22].

## 4. Hypertension as a Possible Risk Factor for Cancer Development

Studies examining the direct associations between HT and the occurrence of cancer have shown considerable inconsistency, despite the shared risk factors between the two conditions. In various observational studies, HT has been suggested as an independent risk factor for renal cell carcinoma (RCC). In a prospective investigation encompassing 48,953 male and 118,191 female participants, it was observed that the relative risk (RR) of developing kidney cancer in individuals with HT was 1.8 for men and 1.9 for women [23]. Examining nearly 300,000 patients over a mean follow-up of 6.2 years, one study revealed a 2.5-fold increased risk of renal cell carcinoma (RCC) associated with elevated systolic BP ≥ 160 mmHg or diastolic BP ≥ 100 mmHg in comparison to lower BP values (systolic BP < 120 mmHg or diastolic BP < 80 mmHg). It is noteworthy that individuals who were both hypertensive and obese showed a heightened risk of developing kidney cancer compared to those with only one of these risk factors [24]. Validation of this association was reinforced through an extensive cohort study comprising nearly 10 million South Korean adults. After an 8-year follow-up, individuals with HT displayed an increased incidence of RCC, recording rates of 20.9 cases per 100,000 person-years, in contrast to 9.2 cases in those without HT [25]. Mechanisms such as HT-induced chronic kidney disease, inflammation, and the upregulation of oncogenic hypoxia-inducible factors and reactive oxygen species are considered to be implicated in the development of RCC among hypertensive individuals [1]. Contrary to RCC, the relationship between HT and the incidence of other malignancies appears to be less evident.

A meta-analysis of observational studies, comprising a pooled population of 1.95 million participants, investigated the relationship between hypertension and colorectal cancer (CRC). The findings indicated a positive association between hypertension and the risk of CRC, with a pooled RR of 1.15 (95% CI: 1.08, 1.23). Specifically, male patients with HT exhibited a 13% increased risk of CRC (95% CI: 1.06, 1.20). Further stratification revealed that the risk of colon cancer and rectal cancer in male patients was 1.17 (95% CI: 1.01, 1.36) and 1.35 (95% CI: 1.04, 1.74), respectively. Intriguingly, no discernible association between HT and the risk of CRC was observed in females [26].

Han et al. conducted a comprehensive meta-analysis of 30 studies aimed at synthesizing evidence on the relationship between HT and the risk of breast cancer. In the subgroup analysis, a positive association between HT and breast cancer incidence was observed specifically among postmenopausal women, with a RR of 1.20 (95% CI: 1.09, 1.31). In contrast, no significant association was identified between HT and the risk of breast cancer among premenopausal women (RR: 0.97; 95% CI: 0.84, 1.12) and in the Asian population (RR: 1.07; 95% CI: 0.94, 1.22) [27].

In an extensive meta-analysis involving 21 studies, a substantial rise in the risk of prostate cancer was identified among individuals with HT, revealing a RR of 1.08 (95% CI 1.02–1.15, *p* = 0.014). Importantly, there was a statistically significant level of heterogeneity among the included studies (*p* < 0.001 for heterogeneity, I^2^ = 72.1%) [28]. After 1 year, Navin and Ioffe undertook a retrospective analysis of 3200 prostate cancer patients aged 51 to 76 years, aiming to ascertain the prevalence of HT. The study population comprised 1388 (43%) African American patients and 1812 (57%) white patients. Among these, HT was identified in 1013 (73%) of African American patients and 1290 (72%) of white patients [29]. Therefore, further prospective studies are warranted to confirm whether HT indeed constitutes a risk factor for prostate cancer.

While potential links between HT and endometrial and liver cancer have been suggested, clear causal relationships are yet to be established. Moreover, other studies indicate that HT is minimally or not associated with several distinct cancer types, encompassing malignancies of the stomach, gallbladder, pancreas, and lung [1].

## 5. Management of Hypertension Related to Anticancer Drugs

As stated before, HT development varies according to cancer type, antineoplastic regimen and associated comorbidities. Because HT is the most common comorbidity in cancer patients, the optimal time to consider HT management is at the time of cancer diagnosis and before the initiation of antineoplastic medication. This will enable the cardio-oncology team to carefully evaluate cancer treatment options, educate the patients regarding BP monitoring and personalizing follow-up visits, according to the CV risk of the patient.

Treatment threshold for HT in cancer patients varies according to the overall CV risk, estimated using established stratification tools, such as SCORE2 in patients aged less than 70 years old and SCORE2-OP in those aged above 70 years old [30,31]. In patients with high CV risk, the accepted threshold for HT treatment before, during and after cancer therapy is ≥130 mmHg for the systolic BP and/or ≥80 mmHg for the diastolic BP. Otherwise, for the rest of the patients, the limit is situated a little higher, at ≥140 mmHg systolic and/or ≥90 mmHg diastolic BP [9].

A comprehensive clinical history, along with physical examination and specific tests should be carried out in order to screen for CV risk factors and end-organ damage [32,33]. Additionally, an electrocardiogram and echocardiogram should be conducted when drugs with potential cardiac toxicity have been prescribed. It is essential to obtain proper BP values prior to anticancer therapy administration in order to prevent the necessity for later cessation or dosage reduction in this treatment due to HT [1]. As a way to acquire trustworthy results, BP measurements should be performed in the clinic [34]. Prior to and throughout therapy, home or ambulatory blood pressure monitoring should be used, if feasible.

The first step in achieving controlled BP involves lifestyle optimization, including smoking cessation, avoiding sedentarism and maintaining adequate physical activity, adopting healthy diets and limiting alcohol consumption. Other comorbidities predisposing to HT development, including untreated sleep apnea, obesity, dyslipidaemia and renal impairment, should be promptly treated and corrected as much as possible, ideally before initiation of anticancer treatment. Correction of these factors should also be considered in patients who develop HT during cancer treatment, before deciding on interruption of the latter.

At the moment, recommendations on the selection of antihypertensive drugs for cancer patients are based upon the risk to develop other cancer therapy-related CV diseases (CTR-CVD), including heart failure. Drugs’ pharmacokinetics and pharmacodynamics, but also comorbidities and possible adverse effects, are all critical factors to keep in mind when selecting antihypertensive medications [1]. Non-dihydropyridine calcium channel blockers (CCB), for instance, should not be taken in conjunction with VEGFI because they could cause cytochrome P450 inhibition, resulting in increased circulating VEGFI levels. Usually, the first-line treatment consists of an angiotensin-converting enzyme inhibitor (ACE-I) or an angiotensin II receptor blocker (ARB). These agents demonstrated efficacy in reducing the risk of CTR-CVD, with the emerging evidence suggesting a significant role of the renin–angiotensin–aldosterone system (RAAS) in the pathogenesis of CTR-CVD. In cancer patients developing grade 2 HT (systolic BP ≥ 160 mmHg and/or diastolic BP ≥ 100 mmHg), initial combination therapy with an ACE-I or ARB and a dihydropyridine CCB is recommended. CCB are expected to be useful in this scenario, considering the involvement of vascular dysfunction in the development of cancer therapy-related HT. The indication to temporarily defer or interrupt cancer therapy should be taken into consideration at persistently elevated BP values, specifically grade 3 HT (systolic BP ≥ 180 mmHg and/or diastolic BP ≥ 110 mmHg) [9]. Cancer therapy, if possible, in reduced doses, can be reinitiated once BP is controlled. In patients with resistant HT, the addition of spironolactone, oral or transdermal nitrates, hydralazine or beta-blockers should be considered. In addition, proper pain and anxiety management should be provided since these variables may directly contribute to BP increase [35]. Sustained monitoring of antihypertensive medication effectiveness is necessary to detect the onset of rebound HT, throughout off-treatment phases or after definitive cessation of antineoplastic therapy. Hypertensive emergencies occurring during cancer treatment, represented by very high BP values associated with acute HT-mediated organ damage, should be immediately managed according to standard acute CV care protocols. The goal is to reduce BP as soon as possible in order to limit the extension of target organ damage.

## 6. Antihypertensive Drugs and Carcinogenesis

The CV benefits of antihypertensive medication are well established, but low adherence is frequently encountered among patients, in part due to concerns about their safety. One raising concern is about the potential carcinogenic effect of antihypertensive drugs, taking into consideration the long-term use of such medication. These concerns were raised initially in 2017 by the US Food and Drug Administration, withdrawing loads of very commonly used antihypertensive drugs, such as losartan, valsartan and irbesartan, after the detection of carcinogenic products of the pharmaceutical process. Traces of N-nitrosodimethylamine and N-nitrosodiethylamine were found in these products, which are known environmental contaminants, classified as probable human carcinogens [36]. Since then, research has focused on the potential carcinogenic effect of antihypertensive drugs, especially with the ongoing evidence suggesting that thiazide diuretics predispose to the development of skin cancer or that ACE-Is are associated with lung cancer.

Diuretics are among the most frequently used antihypertensive classes, often in combination with other drugs. Some studies demonstrated that diuretics increase the risk of developing renal cancer in a dose-dependent fashion [37]. Some toxic metabolites of diuretics, including N-nitroso derivates, have also been advocated. On the other side, this association was not noted in normotensive patients, suggesting that hypertension per se might be a risk factor for renal cell carcinoma, an association that was previously discussed [38]. Most concern focuses on thiazide diuretics, especially hydrochlorothiazide (HCT), which may express carcinogenic effects through their toxic and mutagenic damages in the distal tubules of the nephrons. Additionally, HCT promotes a photosensitizing dermal reaction, causing DNA damage and chronic skin inflammation. These have been hypothesized to be related to skin cancer development, although most observational studies have not assessed the main risk factors for its development, namely exposure to ultraviolet light, presence and number of atypical naevi and fair skin. Therefore, data from observational studies must be interpreted with caution.

A meta-analysis of 9 observational studies investigated the relationship between thiazide drugs and skin cancer. Based on thiazide types, the use of HCT alone or in combination therapies was associated with an increased risk of squamous cell carcinoma, without significant heterogeneity. Long-term use of thiazide (>4.5 years) was found to be associated with an increased risk of squamous cell carcinoma, although high heterogeneity was noted among studies. No association was found between thiazides and basal cell carcinoma [39]. The association between squamous cell carcinoma and HCT was also confirmed in another population-based cohort study from Japan, including >400,000 hypertensive patients [40]. Another study investigated the association between thiazides and malignant melanoma, showing an increased risk for specific histological types of melanoma, namely nodular and lentigo subtypes [41]. Other meta-analysis supported the association between HCT and different skin cancer types [42,43].

ACE-Is and ARBs are widely prescribed for many CVD, including HT, heart failure or after an acute myocardial infarction, due to their proved beneficial effects, decreasing CV and overall mortality. The role of angiotensin in carcinogenesis has been debated, with in vitro studies showing that angiotensin is involved in tumor vascularization and metastasis. Theoretically, ACE-Is and ARBs may have a protective role against cancer, as many tumor cells express type I angiotensin II receptors, but this has not been proven. Previously, in vivo studies showed that blockade of angiotensin receptors type I and stimulation of angiotensin receptors type II promote tumor angiogenesis [44]. ACE-Is may be involved in lung cancer pathogenesis by determining the accumulation of bradykinin in the lung. Bradykinin receptors are located in various tumor tissues, including lung cancer, so that it may directly stimulate tumor development. In addition, ACE-Is lead to accumulation of substance P in lung cancer tissue, which is associated with proliferation and angiogenesis. On the other side, ARBs block the angiotensin receptor type I and have no impact on bradykinin [45,46]. Theoretically, the risk of cancer development is more plausible to be related to ACE-Is more than ARBs, as their mechanism of action differs significantly [47].

Several observational cohort studies suggested an association between ACE-Is and lung cancer. A population-based cohort study, including more than 990,000 patients treated with antihypertensive drugs with a mean follow-up period of 6.4 years, found that the use of ACE-Is was associated with an overall 14% increased risk of lung cancer. This association was related to the duration of use, with a higher risk of 31% among those on ACE-Is for more than 10 years [48]. However, this study is highly criticized, as smoking status seemed not to influence the occurrence of lung cancer in the ACE-I group and smoking duration was not assessed in all populations. Another recent meta-analysis found an association between ACE-Is and an increased risk for lung cancer. However, the results are limited by the significant heterogeneity among the studies, with no consistent stratification of risk factors for cancer, especially smoking status. It is important to note that observational studies are not able to prove causality and that randomized controlled trials (RCTs) are the accepted standard for drug efficacy and safety. With regards to ACE-Is, meta-analyses of RCTs showed no increase in the risk of lung cancer or any type of cancer [49,50].

Data regarding the use of ARBs and the risk of lung cancer are conflicting. In a meta-analysis of randomized controlled trials, Sipahi et al. investigated the occurrence of specific organ cancers in patients treated with ARBs. Most patients received telmisartan as a study drug. ARBs were found to be associated with a modestly increased risk of new cancer diagnosis (absolute risk of 1.2% over an average of 4 years) [51]. The same investigator recently found an association between the cumulative dose of ARB and lung cancer. After 2.5 years of exposure to a maximal daily dose of ARB, the risk of cancer becomes significant [52]. However, data from RCTs showed no increase in the risk of cancer with ARBs [50,53].

CCBs are widespread drugs used in the treatment of various CVD, including HT and angina. Concerns have been raised about the possibility of CCBs to increase the risk of breast cancer. By decreasing intracellular calcium levels, CCBs may prevent the activation of apoptotic pathways and promote tumor development. In a population-based cohort study, the long-term use of CCBs was not associated with an increased risk of breast cancer [54]. These results were supported by other studies, suggesting that there is no relationship between all types of CCBs and tumor development [55,56].

Overall, evidence regarding the association of antihypertensive treatment and cancer remains controversial. Although sub-analyses of RCTs demonstrated no association between antihypertensive treatment and various types of cancer, it should be noted that they were not designed to specifically address this outcome. Moreover, RCTs designed to address this relationship present ethical limitations, so that data are derived from observational studies, which have limited reliability due to their biases. At present, interruption or avoidance of antihypertensive treatment based on oncogenic risks is not justified. Further evidence is needed in order to make general recommendations.

## 7. Conclusions

The interplay between cancer and hypertension is very complex. Although initial efforts in onco-hypertension focused mainly on chemotherapy-induced HT, there is still much to be understood about the molecular mechanisms responsible for this association. HT, either associated with anticancer treatment or with a specific type of cancer, represents a serious burden and can seriously affect outcomes in cancer survivors. Screening algorithms are needed and when HT is detected, prompt treatment and sometimes reduction or even discontinuation of chemotherapy are required. The association between antihypertensive treatment and cancer development is not well established and the results of clinical trials remain inconclusive. Onco-hypertension has emerged as a developing field that focuses on the complex mechanisms between hypertension and cancer, with multidisciplinary teams able to maintain a balance between the efficacy and risks of both antihypertensive drugs and chemotherapy agents. This area of medicine is continuously evolving, incorporated in the vast cardio-oncology field, aiming to gain further knowledge about the association between cancer and CVD.

## 8. Future Research

Future directions for research will need to address molecular mechanisms underlying chemotherapy-induced HT. As currently some molecular mechanisms are only presumed, improved knowledge will enhance the implementation of optimal treatment strategies, including the development of novel antihypertensive agents to address specific cancer pathways. Prospective studies showing the benefits of early detection and treatment of HT in cancer patients are needed, including outcomes on survival and CVD development. Ideally, RCTs enrolling larger number of patients with longer periods of follow-up are required to provide definitive answers. Regarding the association between certain antihypertensive agents and cancer development, larger studies designed to specifically address this outcome are required in order to prove this causality. Finally, in the era of genetic testing, studies should focus on the underlying mechanisms predisposing cancer patients to develop HT, taking into consideration a genetic predisposition.
